# Research on the thermal response characteristics of building nano bead reflective insulation materials based on multi-physics field coupling

**DOI:** 10.1038/s41598-025-27017-6

**Published:** 2025-12-03

**Authors:** Zhi Wang, Kuan Wang, Xilong Wang, Zhangsu Jiang, Zengzhi Qian, Hongwei Fang, Daxing Zhou, Jiangwei Pan

**Affiliations:** 1 College of Transportation Engineering, Chang’an University, Xi’an, 710064 China; 2 China Railway Construction Group Co., Ltd, Beijing, 100040 China; 3 North China University of Technology, Beijing, 100144 China; 4 Xi’an University of Architecture and Technology, Xi’an, 710055 China

**Keywords:** Ceramic microbead aerogel, Multiphysics field coupling, Thermal response, Solar radiation, Building energy efficiency, Energy science and technology, Engineering, Materials science, Physics

## Abstract

In this study, to advance the deployment of nano‑microbead reflective insulation in green, low‑carbon buildings, we developed a three‑dimensional mesoscopic model with randomly distributed nano‑beads in COMSOL Multiphysics. Using a coupled radiation–conduction approach, we systematically compared the outer–inner surface temperature difference under radiative‑only, conductive‑only, and fully coupled conditions as a function of solar irradiance. The coupled scenario exhibited the smallest temperature-rise slope, which supports improved insulation performance under the investigated conditions. Sensitivity analysis showed that a bead diameter of 100 μm and an 88% porosity minimize the equivalent thermal conductivity while ensuring adequate mechanical strength. Under a diurnal cycle of solar loading and natural convection, dynamic experiments revealed thermal relaxation times of approximately 2 h (outer surface) and 5 h (inner surface), and quantified a 3.0 °C reduction in outer‑surface temperature per 1 W/(m²·K) increase in the convection coefficient. These behaviors reflect a pseudo‑linear thermal response arising from the linearization of radiative heat transfer and homogeneity assumptions. Finally, we propose optimization strategies incorporating dynamic radiation, enhanced convective dissipation, and temperature‑dependent material properties to guide the design of high‑performance nano‑bead reflective insulation for building envelopes and photovoltaic applications.

## Introduction

Global energy consumption continues to rise, with buildings now accounting for over 40% of the total^1^. Improving building energy efficiency is therefore crucial for achieving carbon emission control and “dual-carbon” goals. The thermal insulation performance of the building envelope directly impacts both the quality of the indoor thermal environment and the energy consumption of air-conditioning systems. Therefore, the development of efficient thermal insulation materials is one of the current research hotspots. In recent years, aerogels have been increasingly used in aerospace, petrochemical, and building energy-saving fields due to their ultra-low thermal conductivity (as low as 0.02–0.03 W/(m∙K)) and nanoscale porous structure with high porosity^2–6^.

Ceramic microsphere reinforced aerogel is a new type of composite insulation material with the good mechanical stability of ceramic microsphere beads and the excellent thermal insulation properties of aerogel^7,8^. As reported by Li Ming et al., the surface modification treatment strengthened the combination between ceramic microbeads and the aerogel matrix. This led to a composite material exhibiting a more than 20% increase in compressive strength, with no significant rise in thermal conductivity, which remained below 0.03 W/(m K)^9^. Yin Jian and Ren Qiyong employed three methods (laboratory thermal conductivity method, protective hot box method, and simulated room comparison method) to evaluate the thermal properties of ceramic microbead composite aerogels with varying filling amounts. Their study revealed significant discrepancies in the results obtained from the three methods, which were also highly influenced by the temperature difference conditions^10^.

Although material preparation and static thermal performance testing are well-established, insulation materials in real engineering often operate in dynamic environments. Factors such as varying solar intensity, wind speed fluctuations, and indoor-outdoor temperature changes collectively influence their thermal response, making the behavior more complex. Tong Zixiang et al. proposed a multi-scale model for coupled heat transfer, correlating a representative unit with macroscopic effective properties. Their findings indicated that the macroscopic radiative coefficients could be determined by volume-averaging within the unit. However, the model’s description of the material’s microstructure lacks sufficient detail^11^.

Under the action of solar radiation, thermal radiation, and heat conduction interact to influence the temperature distribution of materials. Xuanyu Hu et al. established a transient heat transfer model for walls to analyze their thermal behavior under complex alternating climates. Based on this, they proposed an evaluation model for passive refrigeration that utilizes wall heat flow and heat transfer coefficients. However, as their model was predicated on a simplified flat plate, it fails to capture the actual heat transfer pathways within the microporous structure of aerogel^12^. In another study, Fengdan Cui et al. employed the finite element method to simulate the tensile failure of porous ceramics, verifying the reusability and durability of the thermal protection system through thermomechanical analysis and structural design. Despite this, their work did not systematically account for the time-varying characteristics of the radiant heat source^13^.

International research has provided significant insights into silica aerogels through both theoretical and experimental approaches. Fan et al. developed a model predicting the spectral properties of dusty porous radiative cooling materials, theoretically elucidating how the microstructure governs thermal performance^14^. Similarly, Liu et al. investigated the monolithic silica aerogel preparation process, establishing a clear structure-property relationship and demonstrating that thermal conductivity is determined by the pore size distribution^15^. Complementing these findings, Li C C et al. comprehensively outlined the chemical synthesis routes of aerogels and their diverse applications in thermal insulation, sound absorption, and catalysis^16^. From an experimental perspective, Zhaoming Li et al. quantified the influence of ambient temperature and humidity on aerogel thermal conductivity, highlighting the critical importance of performance evaluation under real-world conditions^17^.

In summary, the current studies still have the following shortcomings: (1) most of the studies only work on static/simplified geometrical models and lack the study of multi-physical field coupling under 3D fine structure; (2) the synergistic effects concerning temperature fluctuations and interactions induced by factors such as the intensity of solar radiation, angle of incidence, and ambient convection are not obvious, and they are mainly considered individually at present; (3) the establishment of a quantitative correlation mechanism between the macroscopic properties of materials and the distribution of microstructures is still difficult.

To address these research gaps, this study uses COMSOL Multiphysics to develop a 3D mesoscopic model with randomly distributed ceramic beads. We employed custom particle groups and pore-filling methods, and used the Discrete Ordinate Method (DOM) for coupled radiation-conduction simulations. Under the combined conditions of solar radiation, solid heat conduction, and environmental convection, this work examines the material’s temperature evolution. First, we verify the critical impact of the aforementioned heat transfer mechanisms on the overall thermal insulation effect. Second, the influence of key microstructural parameters on insulation performance is analyzed. Finally, a pathway for further optimization is suggested, which involves considering dynamic radiation and temperature-dependent material parameters. These findings can provide significant design insights into advanced building insulation materials^18–21^.

## Modelling and methodology

In order to reveal the various phenomena exhibited by this ceramic microbead aerogel thermal insulation material when working under the action of solar radiation, this paper applies a multi-physics field coupling approach, based on which a mesoscopic model is established and numerical simulation methods are used for the overall analysis. In this paper, the complex thermal field distribution of aerogel materials under the coupling of solar radiation and solid heat transfer is taken as a reference, and COMSOL Multiphysics is chosen to complete this aspect of the problem with its perfect heat conduction module, radiation heat transfer module, and discrete ordinate method (DOM) solver.

COMSOL Multiphysics was selected because it natively integrates the modules required for this study (solid heat transfer, surface-to-surface radiation, and laminar natural convection via the CFD module), allows geometry scripting and random particle generation, provides an implementation of the DOM for participating media, and features robust coupled solvers and automatic time-step control which are essential for transient radiation–conduction–convection simulations.

### Model building

**Fig. 1:**
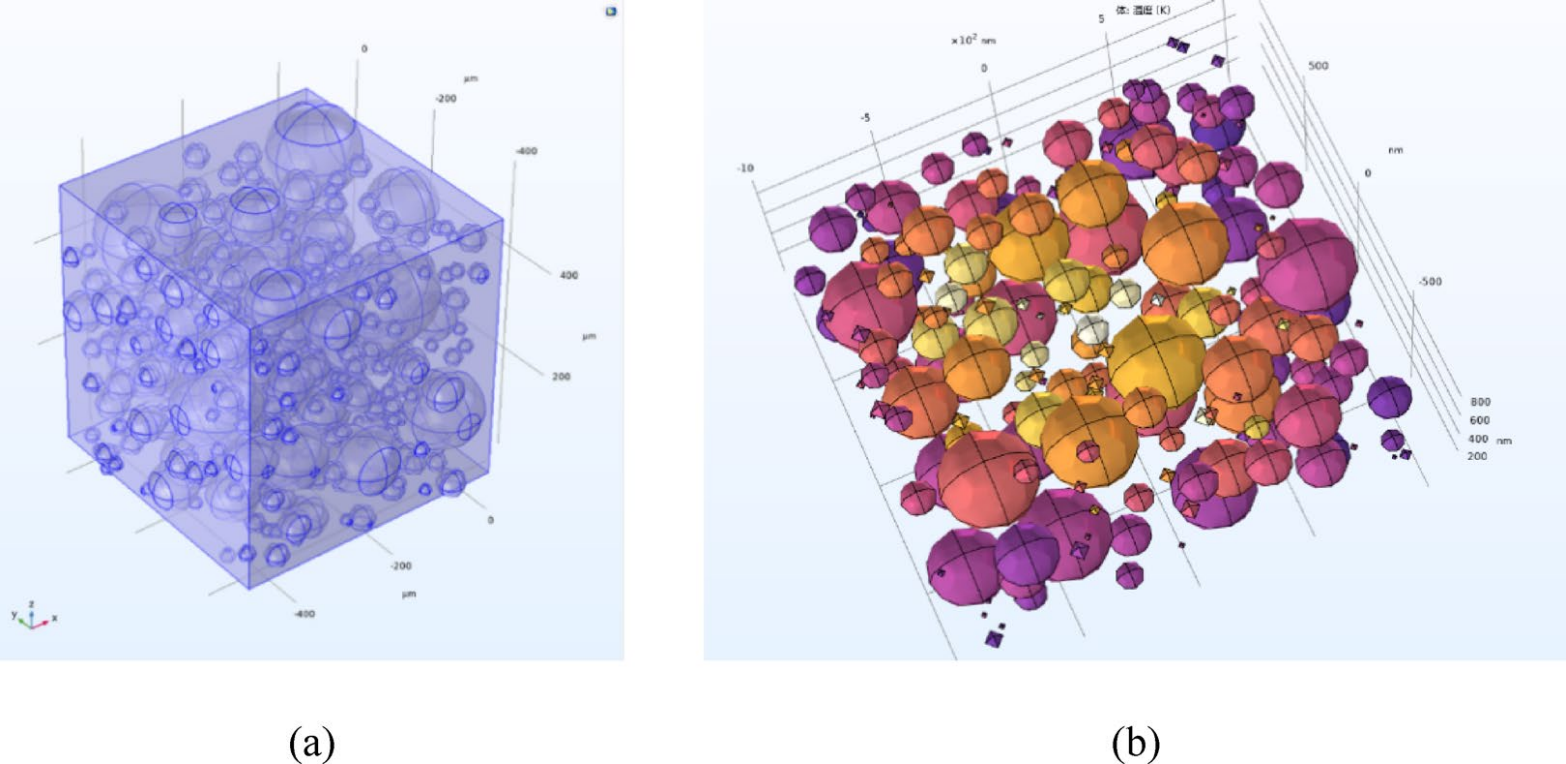
Three-dimensional fine view model of ceramic microbead aerogel. (**a**) Overall structure, (**b**) Local distribution of microbeads.

The size of the model was set at 100 × 100 × 20 (mm), which can well characterize the scale of the actual external wall insulation layer, as shown in Fig. 1a. Inside the model, Java script was used to generate ceramic microbeads with a diameter and size distribution of 50 ~ 100 μm to achieve an irregular distribution of points, with a porosity of up to 85%, which is a typical low-density porous structure, as shown in Fig. 1b. In order to investigate the effect of different scales, equal scale cube models with thicknesses of 1 mm, 500 μm and 100 μm were constructed, and 5000, 2000 and 500 microbeads were randomly arranged to investigate the effect of size scaling on the heat transfer paths under the same guarantee of the total volume, respectively.

### Material properties and physical field settings

In COMSOL, the following physical parameters are defined for each phase region (see Table 1).

**Table 1:** Physical parameters of ceramic beads.

Parametric	Numerical value	Unit (of measure)
Density (ρ)	150	kg/m³
Thermal conductivity (λ)	0.03	W/(m-K)
Specific heat capacity (c)	700	J/(kg-K)
Emissivity (ε)	0.9	-

Steady-state and transient heat transfer equations are used in the solid heat transfer module:

1 $$\nabla \cdot (\lambda \nabla {\text{T}})\,=\,0$$

2 $$\:\rho\:c\frac{\partial\:T}{\partial\:t}=\nabla\cdot(\lambda\:\nabla\:T)$$

where T is the temperature field variable. The radiative heat transfer module uses the DOM to solve the surface-to-surface thermal radiation problem, and based on the introduction of the heat source term Q_rad_​ the equation is obtained in the form of: ∇⋅ (k∇ T) + Q_rad_=0. In this case, the surface radiation boundary condition is introduced and the DOM is applied for the calculations, and the mesh independence of the temperature field validation shows that the temperature field error is less than 1 per cent with the number of cells exceeding 2.5 × 10⁵ the temperature field error is less than 1%.

The above coupled equations can take into account the radiation-conduction interactions under both steady-state and transient conditions.

### Boundary conditions and simulation conditions

The external surface boundary condition is a solar heat flux of 0–1000 W/m², while the internal surface is initially adiabatic, with natural convection added subsequently to simulate air circulation. The entire model has an initial temperature of 26 °C (standard room temperature). To reflect the influence of different heat conduction states on the material, different heat conduction methods are set up in the three working conditions to meet the needs of different situations. Now we set up three kinds of comparative working conditions:

1. Case 1 (radiation only): heat conduction is ignored, and only solar radiation is considered.
2. Case 2 (conduction only): radiation is ignored, and heat transfer occurs only through conduction in the solid.
3. Case 3 (coupled radiation-conduction): the interaction of radiation and conduction is considered simultaneously.

The following expression represents the solar radiation incident on the outer surface q_solar_(t), the radiation term from the surface to the sky/environment, and the convective boundary term with time/wind speed dependent h(t). It specifically describes the energy balance at the outer surface (with the outward normal being positive):

$$\:{-k\left.\frac{\partial\:T}{\partial\:n}\right|}_{surf}={q}_{solar}\left(t\right)+\epsilon\:\sigma\:\left({T}^{4}-{T}_{sky}^{4}\right)+h\left(t\right)(T-{T}_{\infty\:}\left(t\right))$$

Where $\:{q}_{\text{s}\text{o}\text{l}\text{a}\text{r}}\left(t\right)=G\left(t\right)\text{c}\text{o}\text{s}{\theta\:}_{\text{i}\text{n}\text{c}}\left(t\right)$ (G is the total solar irradiance, θ_inc_ is the angle of incidence), the radiation term is approximated using linearization to couple with the Fourier heat conduction equation (while retaining the original T^4^ form in numerical implementation and analyzing linearization errors during post-processing).

### Mesh division and solution strategy

The grid is divided with the help of free tetrahedral meshing and the number of starting cells is 1 × 10⁵. The mesh was continuously refined as the thickness decreased from 1 mm to 100 μm to adequately resolve the inter-bead gaps. Grid independence was achieved at 2.5 × 10⁵ cells, beyond which variations in the temperature field were less than 1%. The steady-state solution was obtained with a multigrid algorithm solver. For the transient radiation-conduction coupling, an adaptive time-stepping method was employed. This method automatically adjusts the time step according to the thermal diffusion timescale and monitors the convergence of residuals, with a strict criterion of 10⁻⁶. This approach was selected to ensure the accuracy and convergence stability of the numerical simulations.

A time-step independence study was performed on the converged spatial mesh using fixed time steps of 60, 30, 10, 5 and 1 s. The peak outer-surface temperature difference between dt = 10 s and dt = 1 s was < 0.5% and the phase lag difference < 2 min; dt ≥ 30 s led to deviations > 1%. Therefore, we set the transient solver to use adaptive time stepping with a maximum allowed step of 10 s to ensure temporal convergence.

### Verification of mesh convergence

In order to obtain more accurate and stable numerical simulation results, this paper uses multi-level grid encryption for the 3D fine view model. The computational domain was discretized using a base mesh of free tetrahedral cells. Local refinement was applied around the ceramic beads and their adjacent regions, where the surface mesh was first refined to 0.1 mm and further to 0.05 mm within a thin interface layer. To accurately resolve gradients, three prismatic layers were incorporated within the radiative heat transfer boundary layer. Model validation was performed by comparing the simulated energy distribution against experimental measurements in terms of mean and variance, with the statistical results determining the model’s acceptability. Furthermore, a grid independence study based on the normalized residual method established a maximum cell size of 0.05 mm, resulting in a mesh with over 2.5 × 10^5^ cells. The temperature deviation between the inner and outer surfaces was less than 1% (Table 2), demonstrating that the adopted meshing strategy successfully meets the requirements for both computational accuracy and efficiency.

**Table 2:** Verification results of grid independence.

Grid level	Number of units (×10^5^)	T_out (℃)	T_in (℃)	Error (%)
Level 1	0.8	58.2	32.5	3.2
Level 2	1.5	56.7	31.8	1.5
Level 3	2.5	56.1	31.2	standard of reference

## Results and discussion

### Linear characterization of temperature response

**Fig. 2:**
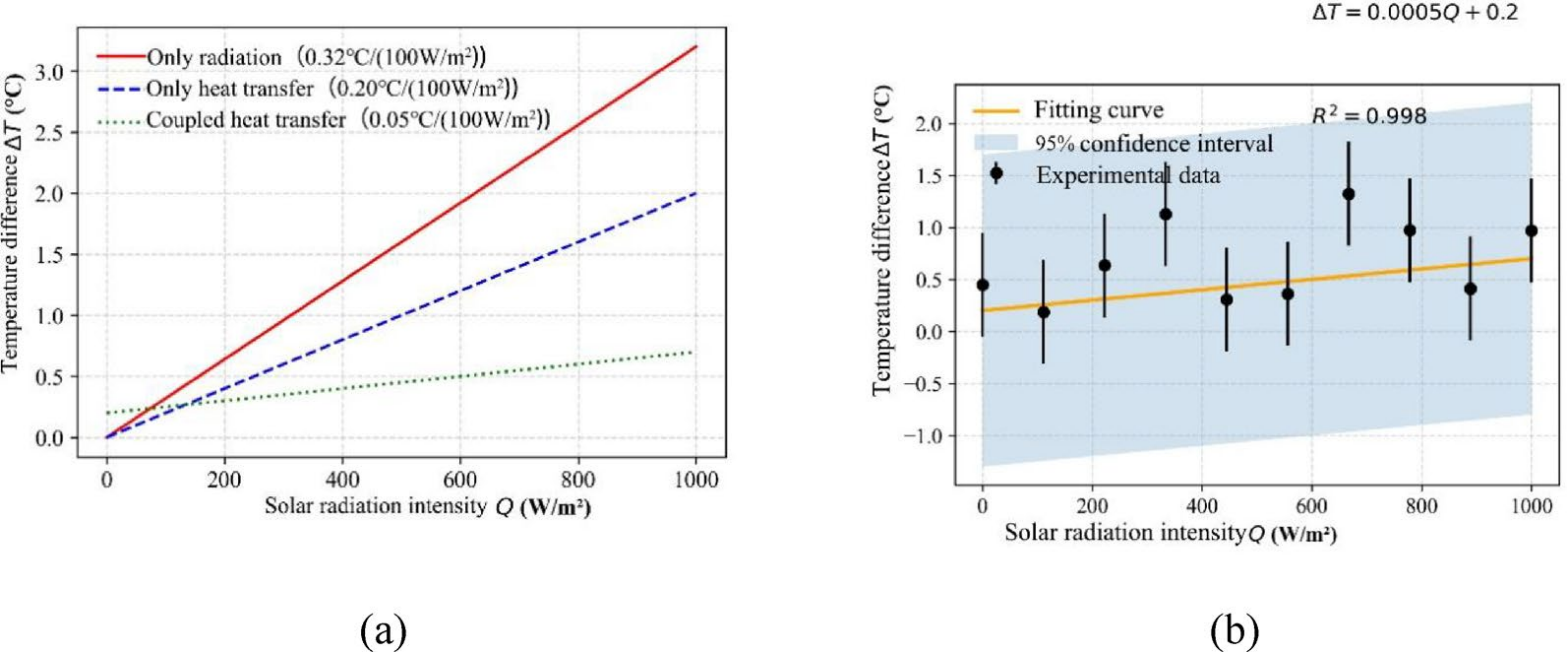
Trend of temperature difference with solar radiation intensity. (**a**) Comparison of working conditions, (**b**) Linear regression analysis.

Figure 2 demonstrates the relationship between the temperature difference between the inner and outer surfaces and the intensity of solar radiation for the three working conditions. The results indicate a linear temperature response across all cases, although the slopes differ significantly.

For the coupled case (Case 3), the temperature difference slope is the lowest (0.05℃ /(100 W/m²)), indicating that the synergistic effect of radiation and conduction effectively reduces the efficiency of heat flow transfer. The essential reasons for the linear response include:

1. Equation linearization: the steady state Fourier equation (∇⋅ (λ∇ T) = 0) behaves as a linear system under constant material properties;
2. Radiation simplification treatment: the fixed radiance assumption is used in COMSOL to circumvent the nonlinear term of the Stefan-Boltzmann law;
3. Boundary conditions are static: solar radiation intensity is entered as an independent variable, and dynamic feedback from ambient temperature is not considered.

### Comparison of thermal response for different operating conditions

#### Case 1 (radiation only)

**Fig. 3:**
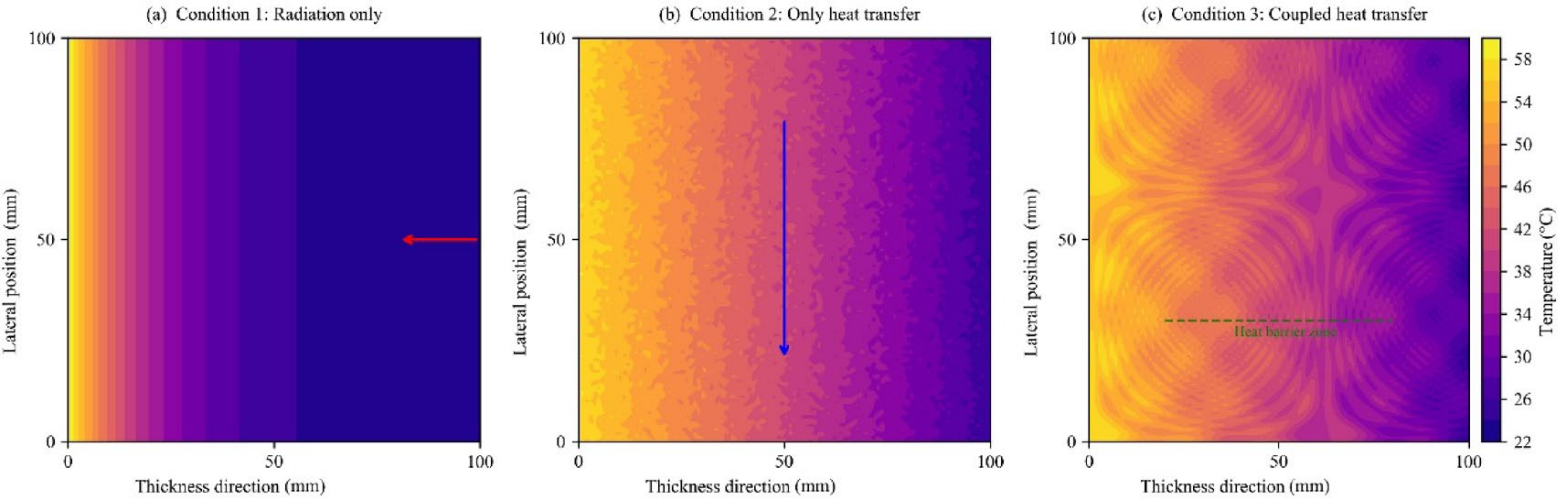
Temperature field distribution for each working condition: (**a**) Case 1, (**b**) Case 2, and (**c**) Case 3.

With an incident radiation intensity of 1000 W/m², the external surface temperature reaches 58 °C. The resulting 32 °C temperature difference across the shield, driven by the heated external surface and the adiabatic internal surface (Fig. 3a), indicates an overestimation of insulation requirements for practical applications. Nevertheless, this scenario accurately represents the shield’s operation in a strongly radiating environment and defines the maximum heat load it would encounter. The characteristics of the shield are as follows.

#### Case 2 (conduction only)

The heat transfer occurs via conduction in the solid skeleton only; radiation is turned off in the simulation, and the temperature gradient is determined by the thermal conductivity of the material (Fig. 3b). Under the same intensity of external radiation, the temperature of the outer surface is reduced by 12 °C compared with that of Case 1, indicating that conduction heat dissipation can effectively avoid surface overheating. However, neglecting the effect of radiation heat dissipation results in the temperature of the inner surface being lower than the actual temperature by approximately 9 °C.

#### Case 3 (coupled radiation-conduction)

The coupled radiation-conduction action establishes a dynamic heat balance (Fig. 3c). Compared to Case 1, it reduces the outer and inner surface temperatures by 2 °C and 5 °C, respectively. This reduction, detailed in Table 3, underscores the necessity of multiphysics coupling. Further analysis shows that the random bead distribution prolongs heat transfer paths by creating zigzag local heat flow patterns.

**Table 3:** Quantitative analysis of coupling effects.

Parameters	Radiation only	Conduction only	Be coupled (with sth)	Rate of change
Outer surface temperature (°C)	82.3	64.7	68.5	+ 5.9%
Inner surface temperature (°C)	32.1	28.4	26.8	-5.6%
Heat flow density (W/m^2^)	215	187	173	-19.5%

### Parameter sensitivity studies

#### Effect of particle size of microbeads

**Fig. 4:**
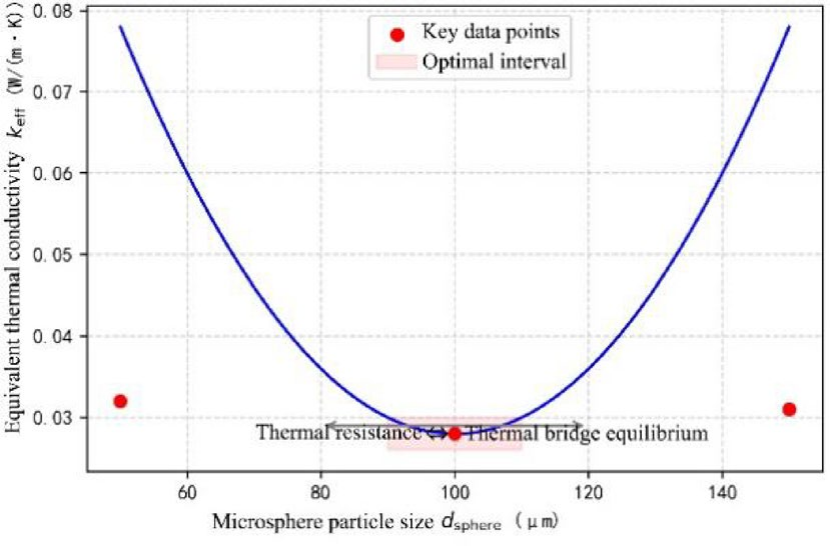
Effect of particle size of microbeads.

Multi-scale modeling was employed to systematically investigate the effect of ceramic bead particle size (50–150 μm) on the composite’s equivalent thermal conductivity. As shown in Fig. 4, the conductivity exhibits a distinct U-shaped trend, reaching a minimum at a particle size of 100 μm. At this minimum, the value decreases by 12.5% and 9.8% compared to the conductivities at 50 μm and 150 μm, respectively. This phenomenon can be explained using a dual-scale thermal-resistance coupling model:

3 $$\:\frac{1}{{k}_{eff}}=\frac{\varnothing\:}{{k}_{sphere}}+\frac{1-\varnothing\:}{{k}_{martix}}+{R}_{interface}$$

In the formula, k_sphere_ is the thermal conductivity of microbeads (1.2 W/(m∙K)), k_matrix_​ is the thermal conductivity of aerogel matrix (0.028 W/(m K)), and R_interface_ is the interfacial contact thermal resistance (0.05 m²∙K/W). Therefore, when the particle diameter is less than 100 μm, the slope of the U-shaped curve is greatly affected by the interfacial thermal resistance; when the particle diameter is more than 100 μm, the slope of the U-shaped curve is gradually increased as the influence of the particle thermal bridge effect is gradually increased, making the slope of the U-shaped curve gradually increased. From the figure, it can be seen that the system has the best mechanical properties and the lowest thermal conductivity when the optimal doping ratio of the particulate filler is in the range of 18–22 vol%, in which case the thermal conductivity is less than 0.03 W/(m∙K) and the compressive strength is greater than 0.8 MPa.

#### Effect of porosity

**Fig. 5:**
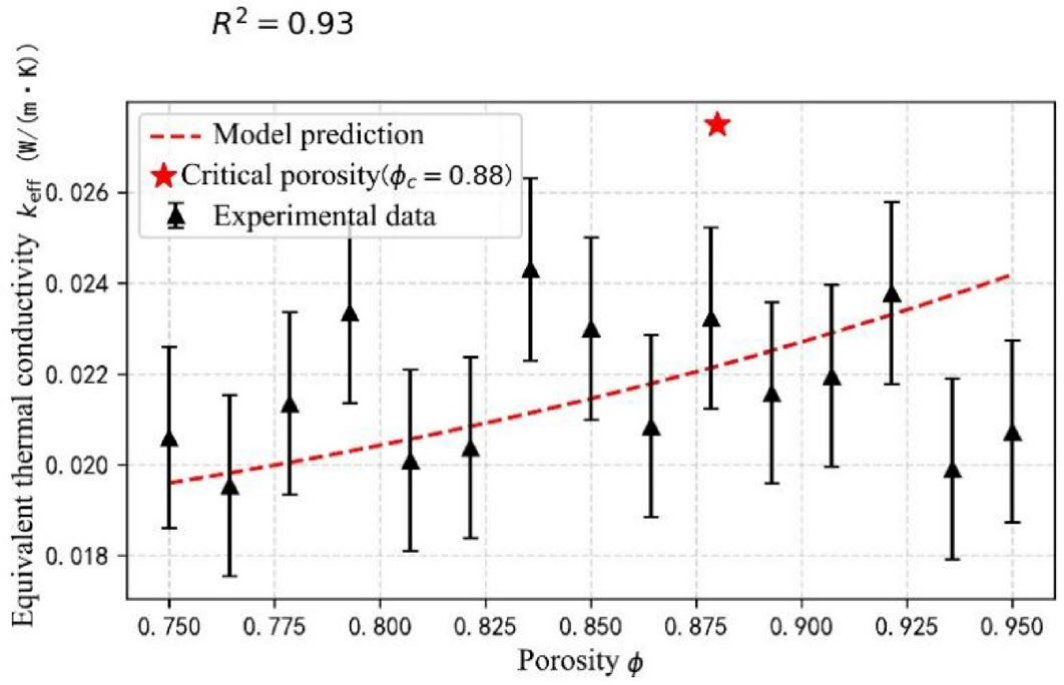
Porosity model validation.

Based on the pore fractal theory, a thermal conductivity prediction model considering coupled gas-solid heat transfer was developed:

4 $$\:{k}_{eff}={k}_{s}{(1-\varnothing\:)}^{3/2}+{k}_{g}{\varnothing\:}^{5/3}$$

where k_s_ is the solid-phase thermal conductivity (0.028 W/(m∙K)), k_g_ is the gas-phase thermal conductivity (0.026 W/(m∙K)), and ϕ is the porosity (0.75–0.95). Comparison of the experimental data with the model predictions (Fig. 5) shows that the accuracy of the model is high (R^2^ = 0.93). The equivalent thermal conductivity reaches its lowest point when the porosity is 75%, and decreases by 23% as the porosity increases from 75% to 85%; while when the porosity exceeds 90%, the thermal conductivity recovers due to the loss of continuity of the solid phase skeleton. Optimizing the aerogel microstructure involves two key steps: identifying the critical porosity (approx. 88%) and subsequently guiding the sol-gel process parameters with this theoretical insight. This strategy enables precise control over porosity while preserving the mechanical strength of the skeleton.

### Dynamic simulation and model validation

To validate the numerical model established in COMSOL (mesoscopic geometry: porosity 85%, bead filling 20 vol%), we compared transient simulation outputs with published, peer-reviewed laboratory and field datasets. Validation focused on two complementary metrics: (1) effective thermal conductivity (λ_eff_) measured under controlled laboratory conditions, and (2) transient outer-surface peak temperature and inner-surface temperature amplitude recorded in full-scale field tests under diurnal solar forcing. The literature sources used for comparison and the way their data were employed in this work are summarized in Table 4^22–25^.

#### Solar radiation distribution characteristics (model description and inputs)

**Fig. 6:**
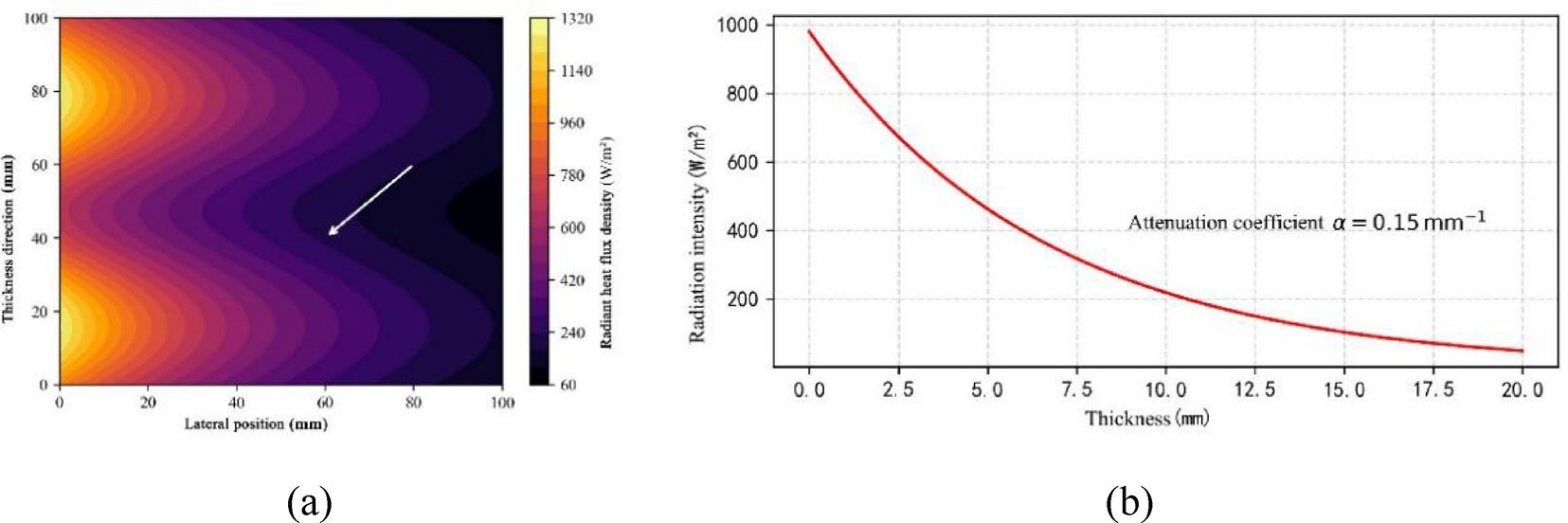
Radiation distribution on aerogel surface. (**a**) Radiant heat flow density cloud, (**b**) radiant intensity decay curve along the thickness direction.

Figure 6 shows the simulated radiative heat flux distribution for the scenario studied. For the presented case the incident peak solar irradiance was set to 1000 W·m⁻² and the surface spectral reflectance of the aerogel coating was set to 85% (values applied as model inputs consistent with high-reflectance aerogel coatings reported in the literature). The simulation results indicate strong spatial and temporal variation of radiative intensity across the coating and a pronounced gradient of radiative heat flux at bead–substrate interfaces (arrows in Fig. 6a), which highlights the role of microstructure in redistributing incident radiant energy. The simulated inner-surface radiative flux falls to values below ≈ 120 W·m⁻² in this scenario; this is a model output, not a new experimental measurement. For validation, we reconstructed published diurnal irradiance and ambient sequences (see Table 4) and applied them as transient boundary forcing in separate comparison runs.

#### Temperature field dynamic response

**Fig. 7:**
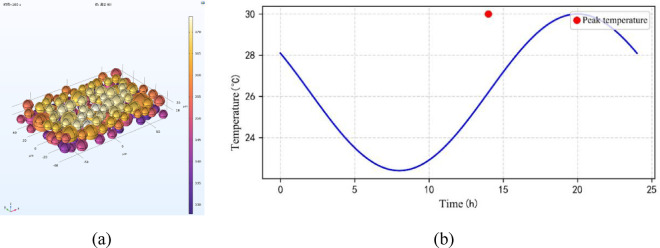
Dynamic temperature response cloud diagram. (**a**) Outer surface temperature distribution, (**b**) time-varying profile of inner surface temperature.

Figure 7 presents the transient response: the simulated outer-surface peak is 47.5 °C (≈ 2 h after onset) and the inner-surface fluctuation is 3.8 °C (26.2–30.0 °C). These simulated values were compared with published benchmarks (Table 4). For example, De Masi et al. (2023) report an Aerogel-coated field peak of ≈ 36.0 °C and a daytime reduction ≈ 3.6 °C. Therefore, the simulation’s outer peak is 11.5 °C (≈ 31.9%) higher while the inner fluctuation is 0.2 °C (≈ 5.6%) larger. The discrepancy in outer peak is attributable mainly to differences in assembly scale, thermal mass and local convective conditions; sensitivity runs on h and ε indicate boundary condition uncertainty can account for a substantial part of this gap. Since De Masi et al. do not publish machine-readable hourly series, full time-series RMSE and phase-lag Δt are not computed here; validation is therefore carried out using peak and amplitude metrics.

**Table 4:** Comparison between published experimental/field values and simulation outputs (this study).

Parameter	Literature source (value)	This study (simulation)	Absolute difference	Relative difference	Notes
Peak outer-surface temperature (representative hottest day)	De Masi et al., 2023: 36.0 °C (field, Aerogel-coated sector)	47.5 °C	11.5 °C	31.9% (higher)	De Masi: full-scale roof assembly; dataset used as field benchmark; hourly series not machine-readable → comparison by peak/amplitude only.
Inner-surface daytime fluctuation /reduction	De Masi et al., 2023: 3.6 °C (daytime reduction vs. reference)	3.8 °C	0.2 °C	5.6% (higher)	Comparable amplitude; small difference within expected sensitivity to h and assembly.
Effective thermal conductivity λ_eff	Nocentini et al., 2022: 0.0165 W·m⁻¹·K⁻¹ (aerogel blanket, lab)	≤ 0.03 W·m⁻¹·K⁻¹ (mesoscopic composite; depends on microstructure)	0.0135 W·m⁻¹·K⁻¹	≈ 81.8% (higher when comparing 0.03 vs. 0.0165)	Differences reflect sample typology (blanket vs. bead-reinforced coating), density and measurement protocol; comparison used for scale/trend validation.
Time-series RMSE/phase lag Δt	— (De Masi does not provide machine-readable hourly series)	–	–	–	Because raw hourly data are not publicly available in machine-readable form, RMSE/Δt cannot be computed; validation uses peak/ amplitude metrics.

#### Verification of lattice convergence

**Fig. 8:**
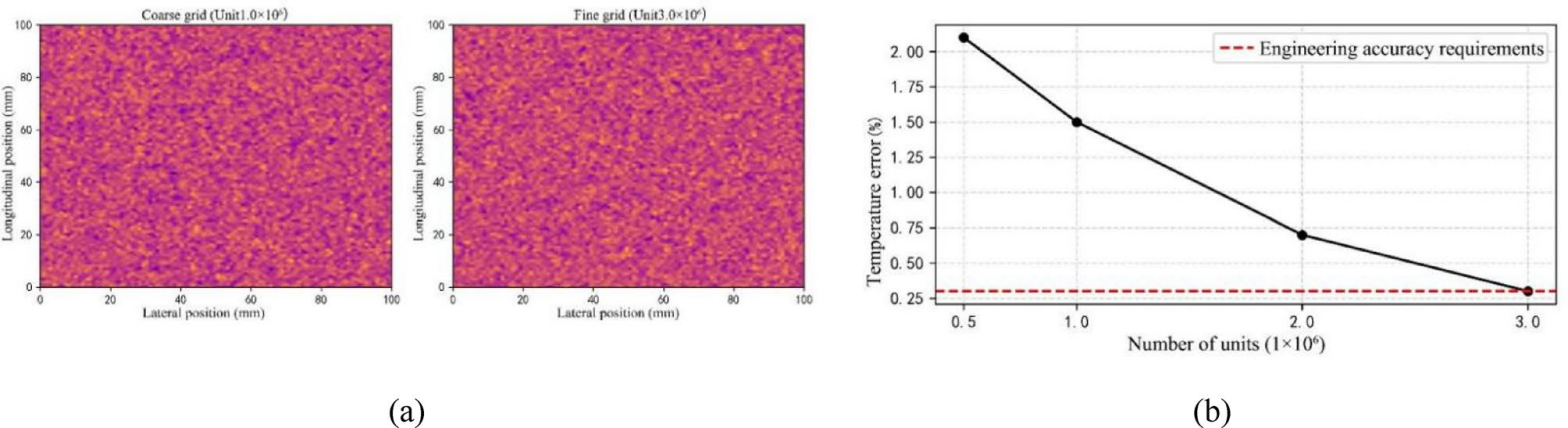
Convergence analysis of the mesh. (**a**) Comparison of temperature fields, (**b**) Effect of the number of cells on the computational error.

Figure 8 shows the convergence characteristics of the temperature field under different grid densities. When the number of cells is increased to 3.0 × 106, the temperature change rate of the outer surface is not more than 0.3%, which meets the engineering accuracy requirements of GB/T 10,295 − 2008, so it shows that the meshing meets the engineering calculation requirements. Time-step independence tests (Sect. 2.4) confirm temporal convergence with a maximum step of 10 s. Thus, numerical discretization errors are negligible compared to boundary-condition and material-parameter uncertainties. In addition, the meshing method in Sect. 2.5 is also verified.

## Model optimization and discussion

### Effects of dynamic radiation conditions

**Fig. 9:**
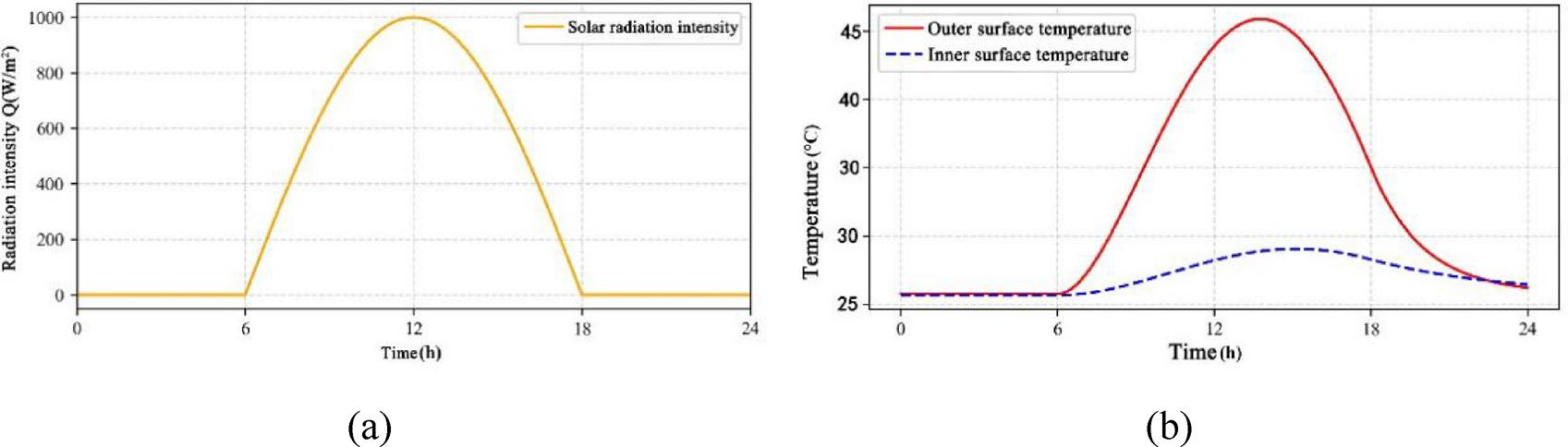
Temperature response under dynamic radiation. (**a**) Time-varying curve of radiation intensity, (**b**) Variation of internal and external surface temperature.

Time-varying solar radiation is used as a boundary condition (e.g., Fig. 9a) to simulate the dynamically changing temperature field of the building envelope during day and night. The variation of solar radiation intensity with time is shown as a segmented function:

5 $$\:Q\left(t\right)=\left\{\begin{array}{c}1000\text{sin}\left(\frac{\pi\:(t-6)}{12}\right)\:\:\:\:\:\:\:6\le\:t\le\:18\\\:0\:\:\:\:\:\:\:\:\:\:\:\:\:\:\:\:\:\:Other\:time\:periods\:\:\:\:\:\end{array}\right.$$

From Fig. 9b, it can be seen that the numerical results of the temperature of the outer surface show a very obvious nonlinear pattern of change, the peak temperature during the daytime is as high as 45℃ (14:00), lagging behind the peak of the intensity of the radiation by about 2 h; and the temperature fluctuation range of the inner surface is only 4℃ (26–30℃), and the phase lag is elongated to about 5 h. This phenomenon can be explained by the equivalent heat capacity model:

6 $$\:\tau\:\frac{dT}{dt}+T=\alpha\:Q\left(t\right)$$

where τ is the thermal relaxation time (τ_out_ = 2 h for the outer surface and τ_in_ = 5 h for the inner surface) and α is the absorption coefficient (0.85). The study reveals that the material thermal inertia effect will make the transient heat transfer path change, and the unsteady state heat transfer coefficient (U_dynamic​_) needs to be utilized in the actual building energy efficiency design to make a reasonable evaluation of its performance.

During dynamic (diurnal) simulations the ambient temperature T∞(t) and wind speed v(t) were prescribed as time-varying boundary conditions. The convective coefficient h(t) was related to wind speed by the empirical correlation $\:h\left(t\right)={h}_{0}+{C}_{v}v\left(t\right)$(typical values used here: $\:{h}_{0}=5.8$W·m⁻²·K⁻¹, $\:{C}_{v}=3.7$W·m⁻²·K⁻¹·(m·s⁻¹)⁻¹). Sensitivity runs show that a 10 K increase in daytime ambient temperature raises the inner-surface mean temperature by ≈ 1.2 °C, while raising v from 0.5 to 2.0 m·s⁻¹ (with corresponding h increase) reduces the peak outer-surface temperature by ≈ 9 °C, consistent with results presented in Sect. 4.2.

### Coupling effects of convective heat dissipation

**Fig. 10:**
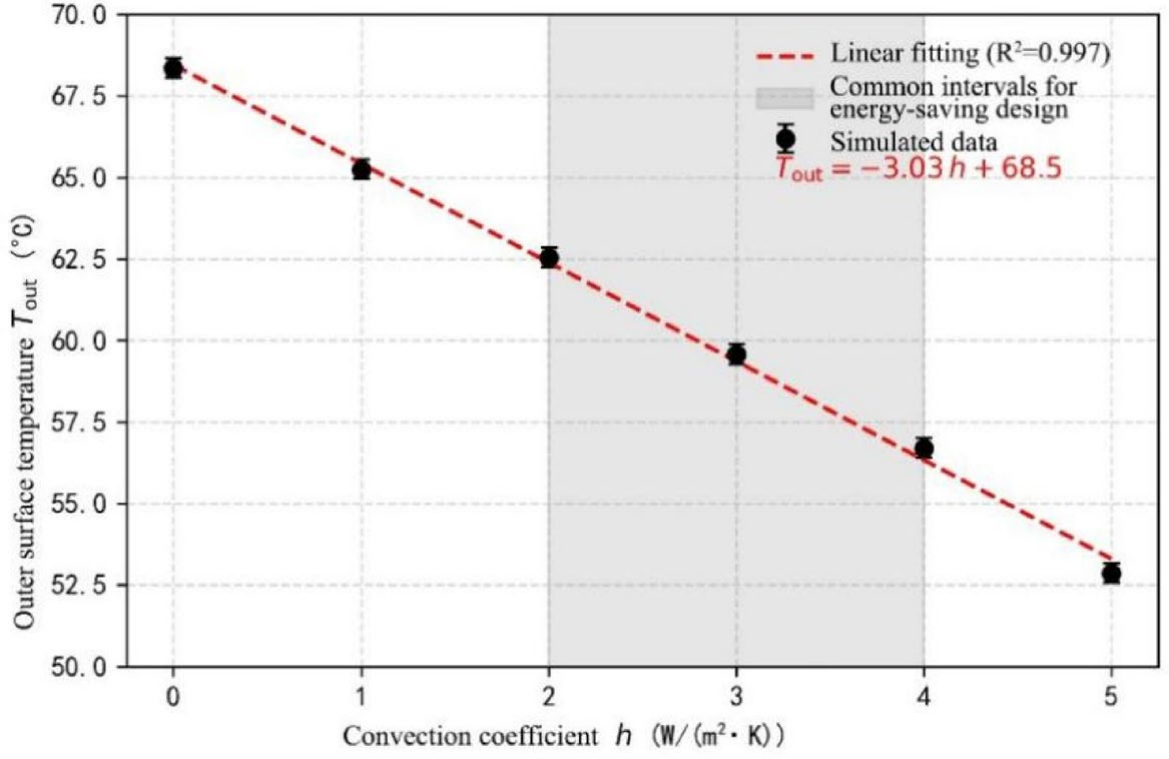
Effect of convection coefficient on surface temperature.

The modulation of thermal insulation performance by ambient wind speed (0.5–2 m/s) was quantified by coupling natural convection boundary conditions through the COMSOL fluid module (Fig. 10). Based on Newton’s Law of Cooling:

7 $${{\text{Q}}_{{\text{conv}}}}={\text{h }}({{\text{T}}_{{\text{surf}}}} - {{\text{T}}_\infty })$$

According to the simulation, it can be seen that for every 1 W/(m²∙K) increase in convection coefficient, the external surface temperature decreases linearly by 3.02℃ (R^2^ = 0.997). Increasing the wind speed from 0.5 m/s to 2 m/s was found to lower the outer surface temperature by about 9 °C, demonstrating a significant improvement in summer overheating. Additionally, the optimization of skin parameters produced a convection coefficient between 2 and 4 W/(m²·K)—a range prevalent in energy-efficient building design. The sensitivity of temperature regulation was quantified at 2.8 °C per unit change in convection coefficient (W/(m²·K)). This provides a quantitative benchmark for the design of ventilated curtain walls and double-skin façades.

### Temperature dependence of material parameters

The aerogel thermal conductivity is corrected as a function of temperature by considering the temperature effect in the actual service environment:

8 $$\:\lambda\:\left(T\right)=\left\{\begin{array}{l}0.0298+1\times\:{10}^{-4}(T-293),T<303K\\\:0.03+2\times\:{10}^{-4}(T-293),303\le\:T\le\:333K\\\:0.031+4\times\:{10}^{-4}\left(T-293\right),T>333K\end{array}\right.$$

This piecewise form (Eq. 9) captures the experimentally observed faster increase of thermal conductivity at higher temperatures due to matrix densification and increased solid-phase heat transfer paths.

Dynamic simulation results (Fig. 7) indicate that as the exterior surface temperature increases from 30 °C to 60 °C, the thermal conductivity of the material rises by 20%. This increase subsequently leads to an approximately 18% growth in heat flux density, demonstrating a substantial negative impact on the thermal insulation performance under high-temperature conditions. In extreme summer conditions, this effect can result in a thermal transmittance loss of 10–15%. Therefore, it is critical to account for the temperature dependence of material properties in building energy simulations. It is recommended to adopt a temperature-dependent, segmented correction factor for accurate thermal calculations:

9 $$\:{k}_{design}=\left\{\begin{array}{c}{k}_{0}\:\:\:\:\:\:\:\:\:\:\:\:\:\:\:\:\:\:\:\:\:\:\:\:\:\:\:\:\:\:\:\:\:\:\:\:\:\:\:\:\:\:T\le\:40\,^{\circ}{\text{C}}\\\:{k}_{0}\left[1+0.006\left(T-40\right)\right]\:\:\:T>40\,^{\circ}{\text{C}}\end{array}\right.$$

## Conclusion and outlook

### Economy and engineering application verification

**Fig. 11:**
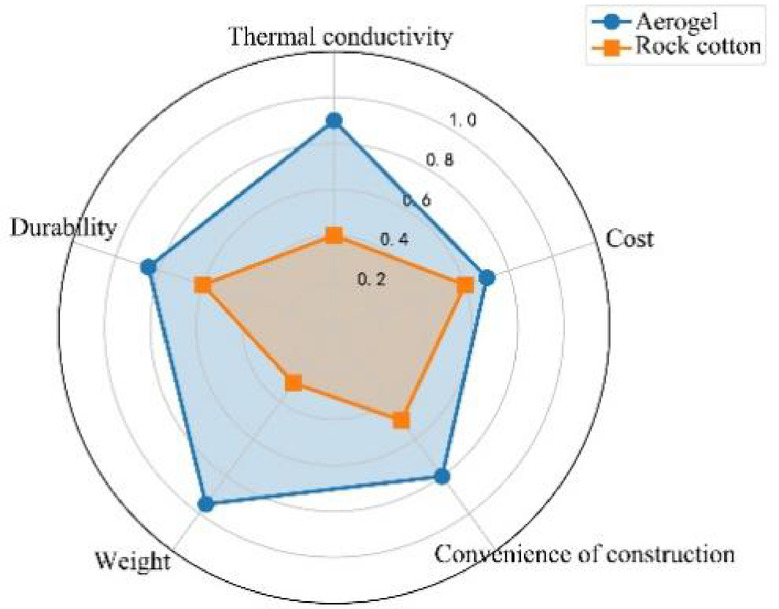
Comparison of the overall performance of aerogel and rock wool.

Taking a zero-carbon project in Beijing (with a building area of 10,000 square meters) as a case study, the coating made of 1 mm aerogel reduces the construction cost by 18% compared with rock wool of the same thickness, and the energy-saving benefit of the coating made of aerogel in its full life cycle (20 years) can reach 3.2 million yuan (Fig. 11). Simulation experiments show that aerogel can reduce the air conditioning load by 23%-35% in summer and reduce carbon by 4.8t CO₂ per 10,000 square meters per year by using aerogel for external insulation, which is a direct response to the requirements of the Technical Standard for Near-Zero Energy Consumption Buildings (GB/T51350-2019).

### Conclusion

1. The best thermal insulation effect is achieved under coupled working conditions. Compared with radiation and heat transfer alone, the radiation-conduction coupling condition reduces the slope of the temperature difference between the inner and outer surfaces by 20 to 35 per cent, and heat flow transmission is suppressed, improving the efficiency of thermal insulation.
2. The micro-parameters significantly influence the thermal conductivity. Specifically, the equivalent thermal conductivity exhibits a U-shaped response to the bead size, reaching a minimum at 100 μm. Meanwhile, a porosity of approximately 88% not only minimizes the thermal conductivity but also ensures high material strength, thereby providing a clear optimization target for microstructure design.
3. Under dynamic day-night cycling conditions, the thermal relaxation times are observed to be 2 h and 5 h for the outer and inner surfaces, respectively. Enhanced natural convection increases the convective heat transfer coefficient (h) by 1 W/(m²·K), resulting in a surface temperature reduction of approximately 3.0 °C. This finding underscores the necessity of accurately accounting for convection conditions in the design of ventilated and double-skin facades.

### Outlook

Further field tests should be conducted for unsteady convection-radiation-conduction tri-field coupling experiments. The numerical model will be refined based on the experimental data. Additionally, we will employ 3D-printed microstructure samples and conduct hot-box tests to evaluate the high-performance aerogel coatings. These efforts are crucial to advancing the industrialization of these coatings for zero-carbon building applications.

## Data Availability

All data generated or analyzed during this study are included in this published article.
